# Potassium *N*-bromo-2-chloro­benzene­sulfonamidate sesquihydrate

**DOI:** 10.1107/S1600536811022136

**Published:** 2011-06-18

**Authors:** B. Thimme Gowda, Sabine Foro, K. Shakuntala

**Affiliations:** a Department of Chemistry, Mangalore University, Mangalagangotri 574 199, Mangalore, India; b Institute of Materials Science, Darmstadt University of Technology, Petersenstrasse 23, D-64287 Darmstadt, Germany

## Abstract

In the structure of the title compound, K^+^·C_6_H_4_BrClNO_2_S^−^·1.5H_2_O, the K^+^ ion is hepta­coordinated by three O atoms from water mol­ecules and by four sulfonyl O atoms of *N*-bromo-2-chloro­benzene­sulfonamidate anions. The S—N distance of 1.582 (4) Å is consistent with an S=N double bond. The crystal structure is stabilized by inter­molecular O—H⋯Br and O—H⋯N hydrogen bonds. The asymmetric unit consits of one potassium cation, one *N*-bromo-2-chloro­benzene­sulfonamidate anion and one water mol­ecule in general positions and one water mol­ecule located on a twofold rotation axis.

## Related literature

For preparation of *N*-haloaryl­sulfonamides, see: Usha & Gowda (2006 ▶). For our study of the effect of substituents on the structures of *N*-haloaryl­sulfonamides, see: Gowda *et al.* (2010 ▶, 2011*a* ▶,*b* ▶). For related structures, see: George *et al.* (2000 ▶); Olmstead & Power (1986 ▶).
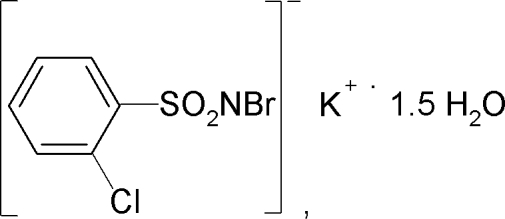


## Experimental

### 

#### Crystal data

K^+^·C_6_H_4_BrClNO_2_S^−^·1.5H_2_O
*M* *_r_* = 335.65
Orthorhombic, 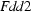

*a* = 12.343 (2) Å
*b* = 52.066 (6) Å
*c* = 6.942 (1) Å
*V* = 4461.3 (11) Å^3^
*Z* = 16
Mo *K*α radiation
μ = 4.47 mm^−1^
*T* = 293 K
0.44 × 0.40 × 0.20 mm

#### Data collection

Oxford Diffraction Xcalibur diffractometer with a Sapphire CCD detector
Absorption correction: multi-scan (*CrysAlis RED*; Oxford Diffraction, 2009 ▶) *T*_min_ = 0.244, *T*_max_ = 0.468
4075 measured reflections
1909 independent reflections
1841 reflections with *I* > 2σ(*I*)
*R*_int_ = 0.026

#### Refinement

*R*[*F* ^2^ > 2σ(*F* ^2^)] = 0.026
*wR*(*F* ^2^) = 0.067
*S* = 1.06
1909 reflections
141 parameters
4 restraints
H atoms treated by a mixture of independent and constrained refinement
Δρ_max_ = 0.42 e Å^−3^
Δρ_min_ = −0.52 e Å^−3^
Absolute structure: Flack (1983 ▶), 671 Friedel pairs
Flack parameter: 0.019 (9)

### 

Data collection: *CrysAlis CCD* (Oxford Diffraction, 2009 ▶); cell refinement: *CrysAlis RED* (Oxford Diffraction, 2009 ▶); data reduction: *CrysAlis RED*; program(s) used to solve structure: *SHELXS97* (Sheldrick, 2008 ▶); program(s) used to refine structure: *SHELXL97* (Sheldrick, 2008 ▶); molecular graphics: *PLATON* (Spek, 2009 ▶); software used to prepare material for publication: *SHELXL97*.

## Supplementary Material

Crystal structure: contains datablock(s) I, global. DOI: 10.1107/S1600536811022136/nc2232sup1.cif

Structure factors: contains datablock(s) I. DOI: 10.1107/S1600536811022136/nc2232Isup2.hkl

Additional supplementary materials:  crystallographic information; 3D view; checkCIF report

## Figures and Tables

**Table 1:** Hydrogen-bond geometry (Å, °)

*D*—H⋯*A*	*D*—H	H⋯*A*	*D*⋯*A*	*D*—H⋯*A*
O3—H31⋯N1^i^	0.80 (2)	2.16 (2)	2.937 (4)	164 (5)
O3—H32⋯Br1^ii^	0.80 (2)	2.83 (3)	3.574 (3)	156 (4)
O4—H41⋯N1^iii^	0.82 (2)	2.13 (3)	2.905 (4)	157 (5)

Symmetry codes: (i) 
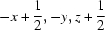
; (ii) 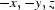; (iii) 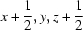.

**Fig. 1.**
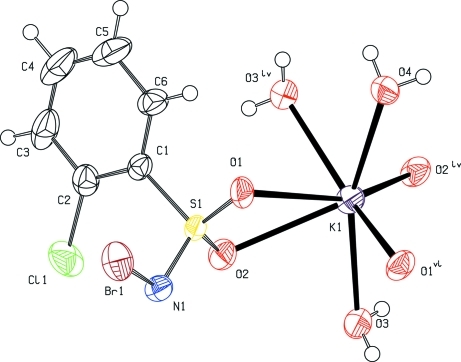
Molecular structure of the title compound, showing the atom labelling scheme for the asymmetric unit and extended to show the coordination geometry for the K+. The displacement ellipsoids are drawn at the 50% probability level. The H atoms are represented as small spheres of arbitrary radii. Symmetry codes: (i) x +1/2, y , z -1/2; (ii) - x +1/2, - y , z -1/2".

**Fig. 2.**
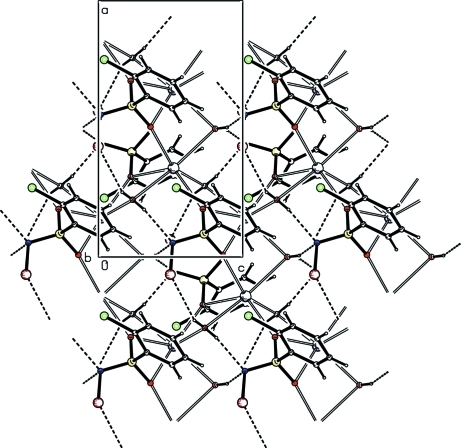
Molecular packing of the title compound with hydrogen bonding shown as dashed lines.

## supplementary crystallographic information

### Comment

To explore the effect of replacing sodium by potassium on the solid state structures of *N*-haloarylsulfonamidates (Gowda *et al.*, 2011**a*,b*), the structure of potassium *N*-bromo-2-chloro-benzenesulfonamidate sesquihydrate (I) has been determined (Fig. 1). The structure of I resembles those of sodium *N*-bromo-2-chloro-benzenesulfonamidate sesquihydrate (II) (Gowda *et al.*, 2011*b*), sodium *N*-chloro-2-chloro- benzenesulfonamidate sesquihydrate (III)(Gowda *et al.*, 2010), potassium *N*-chloro-4-chloro-benzenesulfonamidate monohydrate (IV)(Gowda *et al.*, 2011*a*), and other sodium *N*-chloro- arylsulfonamidates (George *et al.*, 2000; Olmstead & Power, 1986).

In the structure of the title compound the K^+^ ion is hepta coordinated by three O atoms from water molecules and by four sulfonyl O atoms of *N*-bromo-2-chloro-benzenesulfonamide anions. The replacement of Na^+^ by K^+^ changes the coordination from hexa to hepta coordination (Gowda *et al.*, 2011*b*).

The S—N distance of 1.582 (4)Å is consistent with an S—N double bond and is in agreement with the observed values of 1.579 (6) Å in (II), 1.588 (2) Å in (III) and 1.588 (2) Å in (IV)

In the crystal structure two-dimensional polymeric layer are found that are located parallel to the *ac* plane (Fig. 2). The molecular packing is stabilized by O3—H31···N1, O3—H32···Br1 and O4—H41···N1 hydrogen bonds (Table 1).

### Experimental

The title compound was prepared according to the literature method (Usha & Gowda, 2006). The purity of the compound was checked by determining its melting point (176 °). It was characterized by recording its infrared and NMR spectra. Yellow prisms of the title compound used in X-ray diffraction studies were obtained from its aqueous solution at room temperature.

### Refinement

The H atoms bound to O atoms were located in difference map and later restrained to O—H = 0.82 (2) Å. The other H atoms were positioned with idealized geometry using a riding model with C—H = 0.93 Å. All H atoms were refined with isotropic displacement parameters (set to 1.2 times of the *U*_eq_ of the parent atom).

The absolute structure was determined on the basis of 671 Friedel pairs pairs.

### Crystal data

**Table:**

K^+^·C_6_H_4_BrClNO_2_S^−^·1.5H_2_O	*F*(000) = 2640
*M**_r_* = 335.65	*D*_x_ = 1.999 Mg m^−^^3^
Orthorhombic, *F**d**d*2	Mo *K*α radiation, λ = 0.71073 Å
Hall symbol: F 2 -2d	Cell parameters from 2515 reflections
*a* = 12.343 (2) Å	θ = 2.9–27.9°
*b* = 52.066 (6) Å	µ = 4.47 mm^−^^1^
*c* = 6.942 (1) Å	*T* = 293 K
*V* = 4461.3 (11) Å^3^	Prism, yellow
*Z* = 16	0.44 × 0.40 × 0.20 mm

### Data collection

**Table:**

Oxford Diffraction Xcalibur diffractometer with a Sapphire CCD detector	1909 independent reflections
Radiation source: fine-focus sealed tube	1841 reflections with *I* > 2σ(*I*)
graphite	*R*_int_ = 0.026
Rotation method data acquisition using ω scans.	θ_max_ = 26.4°, θ_min_ = 3.1°
Absorption correction: multi-scan (*CrysAlis RED*; Oxford Diffraction, 2009)	*h* = −8→15
*T*_min_ = 0.244, *T*_max_ = 0.468	*k* = −56→64
4075 measured reflections	*l* = −8→6

### Refinement

**Table:**

Refinement on *F*^2^	Secondary atom site location: difference Fourier map
Least-squares matrix: full	Hydrogen site location: inferred from neighbouring sites
*R*[*F*^2^ > 2σ(*F*^2^)] = 0.026	H atoms treated by a mixture of independent and constrained refinement
*wR*(*F*^2^) = 0.067	*w* = 1/[σ^2^(*F*_o_^2^) + (0.0419*P*)^2^ + 7.9401*P*] where *P* = (*F*_o_^2^ + 2*F*_c_^2^)/3
*S* = 1.06	(Δ/σ)_max_ = 0.002
1909 reflections	Δρ_max_ = 0.42 e Å^−^^3^
141 parameters	Δρ_min_ = −0.52 e Å^−^^3^
4 restraints	Absolute structure: Flack (1983), 671 Friedel pairs
Primary atom site location: structure-invariant direct methods	Flack parameter: 0.019 (9)

### Special details

**Table:**

Experimental. CrysAlis RED (Oxford Diffraction, 2009) Empirical absorption correction using spherical harmonics, implemented in SCALE3 ABSPACK scaling algorithm.
Geometry. All e.s.d.'s (except the e.s.d. in the dihedral angle between two l.s. planes) are estimated using the full covariance matrix. The cell e.s.d.'s are taken into account individually in the estimation of e.s.d.'s in distances, angles and torsion angles; correlations between e.s.d.'s in cell parameters are only used when they are defined by crystal symmetry. An approximate (isotropic) treatment of cell e.s.d.'s is used for estimating e.s.d.'s involving l.s. planes.
Refinement. Refinement of *F*^2^ against ALL reflections. The weighted *R*-factor *wR* and goodness of fit *S* are based on *F*^2^, conventional *R*-factors *R* are based on *F*, with *F* set to zero for negative *F*^2^. The threshold expression of *F*^2^ > σ(*F*^2^) is used only for calculating *R*-factors(gt) *etc*. and is not relevant to the choice of reflections for refinement. *R*-factors based on *F*^2^ are statistically about twice as large as those based on *F*, and *R*- factors based on ALL data will be even larger.

### Fractional atomic coordinates and isotropic or equivalent isotropic displacement parameters (Å^2^)

**Table:**

	*x*	*y*	*z*	*U*_iso_*/*U*_eq_	
C1	0.1277 (3)	0.07221 (6)	0.8359 (5)	0.0279 (7)	
C2	0.2039 (3)	0.08804 (7)	0.7551 (6)	0.0366 (8)	
C3	0.2302 (4)	0.11022 (8)	0.8473 (8)	0.0527 (12)	
H3	0.2811	0.1213	0.7935	0.063*	
C4	0.1824 (4)	0.11628 (8)	1.0178 (9)	0.0620 (14)	
H4	0.2017	0.1315	1.0788	0.074*	
C5	0.1081 (4)	0.10099 (9)	1.1015 (8)	0.0568 (13)	
H5	0.0766	0.1055	1.2184	0.068*	
C6	0.0802 (3)	0.07872 (7)	1.0103 (7)	0.0383 (8)	
H6	0.0291	0.0678	1.0655	0.046*	
Br1	−0.06844 (3)	0.067404 (8)	0.49856 (7)	0.04826 (13)	
Cl1	0.26996 (9)	0.08136 (2)	0.54056 (16)	0.0559 (3)	
K1	0.34639 (6)	0.007514 (14)	0.52051 (13)	0.03396 (18)	
N1	0.0549 (2)	0.04684 (5)	0.5150 (5)	0.0326 (6)	
O1	0.0081 (2)	0.03366 (5)	0.8608 (4)	0.0389 (6)	
O2	0.1870 (2)	0.02816 (5)	0.7239 (5)	0.0386 (6)	
O3	0.2768 (2)	−0.03195 (5)	0.7376 (5)	0.0412 (6)	
H31	0.315 (3)	−0.0386 (9)	0.817 (6)	0.049*	
H32	0.247 (4)	−0.0416 (8)	0.666 (6)	0.049*	
O4	0.5000	0.0000	0.8095 (6)	0.0466 (10)	
H41	0.505 (4)	0.0112 (7)	0.892 (6)	0.056*	
S1	0.09016 (6)	0.043209 (13)	0.73226 (12)	0.02646 (17)	

### Atomic displacement parameters (Å^2^)

**Table:**

	*U*^11^	*U*^22^	*U*^33^	*U*^12^	*U*^13^	*U*^23^
C1	0.0302 (16)	0.0232 (15)	0.0302 (17)	0.0029 (13)	−0.0063 (14)	0.0003 (13)
C2	0.0397 (18)	0.0328 (16)	0.037 (2)	−0.0047 (13)	−0.0093 (19)	0.0048 (18)
C3	0.060 (3)	0.030 (2)	0.068 (3)	−0.0135 (19)	−0.023 (2)	0.004 (2)
C4	0.078 (3)	0.032 (2)	0.075 (4)	0.001 (2)	−0.028 (3)	−0.019 (2)
C5	0.073 (3)	0.044 (2)	0.053 (3)	0.019 (2)	−0.013 (2)	−0.022 (2)
C6	0.048 (2)	0.0327 (17)	0.035 (2)	0.0119 (15)	−0.0027 (19)	−0.0056 (18)
Br1	0.0399 (2)	0.0498 (2)	0.0551 (3)	0.00658 (16)	−0.0060 (2)	0.0135 (2)
Cl1	0.0568 (6)	0.0659 (7)	0.0449 (6)	−0.0265 (5)	0.0108 (5)	0.0044 (5)
K1	0.0342 (4)	0.0329 (4)	0.0348 (4)	0.0038 (3)	0.0060 (3)	0.0016 (3)
N1	0.0353 (14)	0.0298 (13)	0.0328 (16)	0.0011 (11)	0.0023 (14)	−0.0036 (14)
O1	0.0466 (15)	0.0280 (12)	0.0421 (16)	−0.0063 (11)	0.0119 (12)	0.0061 (11)
O2	0.0422 (13)	0.0306 (11)	0.0429 (16)	0.0119 (10)	0.0037 (12)	0.0029 (12)
O3	0.0528 (16)	0.0311 (13)	0.0396 (16)	−0.0014 (11)	−0.0041 (15)	−0.0001 (13)
O4	0.070 (3)	0.038 (2)	0.031 (2)	−0.0152 (19)	0.000	0.000
S1	0.0315 (4)	0.0186 (3)	0.0293 (4)	0.0007 (3)	0.0042 (3)	0.0006 (3)

### Geometric parameters (Å, °)

**Table:**

C1—C2	1.371 (5)	K1—O3^ii^	2.790 (3)
C1—C6	1.387 (6)	K1—O2^ii^	2.803 (3)
C1—S1	1.736 (3)	K1—O1^ii^	3.008 (3)
C2—C3	1.360 (6)	K1—S1^ii^	3.4047 (11)
C2—Cl1	1.733 (5)	K1—H32	3.01 (4)
C3—C4	1.360 (8)	N1—S1	1.582 (4)
C3—H3	0.9300	O1—S1	1.438 (3)
C4—C5	1.346 (8)	O1—K1^iii^	2.659 (3)
C4—H4	0.9300	O1—K1^iv^	3.008 (3)
C5—C6	1.365 (6)	O2—S1	1.431 (3)
C5—H5	0.9300	O2—K1^iv^	2.803 (3)
C6—H6	0.9300	O3—K1^iv^	2.790 (3)
Br1—N1	1.864 (3)	O3—H31	0.804 (19)
K1—O2	2.649 (3)	O3—H32	0.796 (19)
K1—O1^i^	2.659 (3)	O4—K1^v^	2.788 (3)
K1—O3	2.689 (3)	O4—H41	0.819 (19)
K1—O4	2.788 (3)	S1—K1^iv^	3.4047 (11)
			
C2—C1—C6	120.0 (3)	O1^i^—K1—S1^ii^	88.82 (6)
C2—C1—S1	122.4 (3)	O3—K1—S1^ii^	79.06 (7)
C6—C1—S1	117.5 (3)	O4—K1—S1^ii^	99.07 (5)
C3—C2—C1	118.8 (4)	O3^ii^—K1—S1^ii^	93.74 (7)
C3—C2—Cl1	117.5 (3)	O2^ii^—K1—S1^ii^	24.26 (5)
C1—C2—Cl1	123.6 (3)	O1^ii^—K1—S1^ii^	24.95 (5)
C2—C3—C4	120.2 (4)	O2—K1—H32	82.2 (9)
C2—C3—H3	119.9	O1^i^—K1—H32	151.7 (9)
C4—C3—H3	119.9	O3—K1—H32	14.7 (6)
C5—C4—C3	122.3 (4)	O4—K1—H32	85.2 (7)
C5—C4—H4	118.9	O3^ii^—K1—H32	113.7 (7)
C3—C4—H4	118.9	O2^ii^—K1—H32	67.9 (6)
C4—C5—C6	118.3 (5)	O1^ii^—K1—H32	76.2 (9)
C4—C5—H5	120.9	S1^ii^—K1—H32	68.4 (8)
C6—C5—H5	120.9	S1—N1—Br1	110.60 (17)
C5—C6—C1	120.4 (4)	S1—O1—K1^iii^	164.64 (17)
C5—C6—H6	119.8	S1—O1—K1^iv^	93.13 (13)
C1—C6—H6	119.8	K1^iii^—O1—K1^iv^	85.96 (7)
O2—K1—O1^i^	124.89 (9)	S1—O2—K1	149.8 (2)
O2—K1—O3	76.94 (8)	S1—O2—K1^iv^	102.12 (15)
O1^i^—K1—O3	149.87 (9)	K1—O2—K1^iv^	103.41 (8)
O2—K1—O4	100.27 (10)	K1—O3—K1^iv^	102.72 (8)
O1^i^—K1—O4	82.02 (8)	K1—O3—H31	122 (4)
O3—K1—O4	72.95 (7)	K1^iv^—O3—H31	92 (4)
O2—K1—O3^ii^	77.62 (10)	K1—O3—H32	106 (4)
O1^i^—K1—O3^ii^	83.23 (9)	K1^iv^—O3—H32	118 (4)
O3—K1—O3^ii^	124.66 (5)	H31—O3—H32	116 (5)
O4—K1—O3^ii^	160.21 (6)	K1—O4—K1^v^	87.96 (13)
O2—K1—O2^ii^	123.44 (5)	K1—O4—H41	117 (4)
O1^i^—K1—O2^ii^	98.26 (9)	K1^v^—O4—H41	123 (4)
O3—K1—O2^ii^	81.86 (9)	O2—S1—O1	115.10 (17)
O4—K1—O2^ii^	122.39 (7)	O2—S1—N1	104.89 (17)
O3^ii^—K1—O2^ii^	72.84 (8)	O1—S1—N1	116.06 (16)
O2—K1—O1^ii^	158.24 (8)	O2—S1—C1	105.67 (17)
O1^i^—K1—O1^ii^	76.30 (9)	O1—S1—C1	103.37 (17)
O3—K1—O1^ii^	81.53 (9)	N1—S1—C1	111.42 (15)
O4—K1—O1^ii^	76.07 (7)	O2—S1—K1^iv^	53.61 (12)
O3^ii^—K1—O1^ii^	113.00 (8)	O1—S1—K1^iv^	61.92 (11)
O2^ii^—K1—O1^ii^	49.09 (7)	N1—S1—K1^iv^	135.78 (10)
O2—K1—S1^ii^	143.01 (7)	C1—S1—K1^iv^	111.69 (11)
			
C6—C1—C2—C3	−1.1 (5)	O1^i^—K1—O4—K1^v^	−41.28 (6)
S1—C1—C2—C3	−178.8 (3)	O3—K1—O4—K1^v^	121.76 (7)
C6—C1—C2—Cl1	178.7 (3)	O3^ii^—K1—O4—K1^v^	−83.5 (3)
S1—C1—C2—Cl1	0.9 (5)	O2^ii^—K1—O4—K1^v^	53.47 (8)
C1—C2—C3—C4	0.8 (6)	O1^ii^—K1—O4—K1^v^	36.50 (6)
Cl1—C2—C3—C4	−178.9 (3)	S1^ii^—K1—O4—K1^v^	46.24 (2)
C2—C3—C4—C5	−0.2 (7)	K1—O2—S1—O1	139.5 (3)
C3—C4—C5—C6	−0.2 (7)	K1^iv^—O2—S1—O1	−7.7 (2)
C4—C5—C6—C1	0.0 (6)	K1—O2—S1—N1	10.7 (4)
C2—C1—C6—C5	0.7 (6)	K1^iv^—O2—S1—N1	−136.49 (13)
S1—C1—C6—C5	178.5 (3)	K1—O2—S1—C1	−107.1 (3)
O1^i^—K1—O2—S1	71.6 (4)	K1^iv^—O2—S1—C1	105.67 (15)
O3—K1—O2—S1	−131.4 (3)	K1—O2—S1—K1^iv^	147.2 (4)
O4—K1—O2—S1	159.0 (3)	K1^iii^—O1—S1—O2	−79.1 (7)
O3^ii^—K1—O2—S1	−1.0 (3)	K1^iv^—O1—S1—O2	7.01 (19)
O2^ii^—K1—O2—S1	−60.5 (3)	K1^iii^—O1—S1—N1	43.9 (7)
O1^ii^—K1—O2—S1	−122.9 (3)	K1^iv^—O1—S1—N1	130.04 (12)
S1^ii^—K1—O2—S1	−80.6 (3)	K1^iii^—O1—S1—C1	166.2 (6)
O1^i^—K1—O2—K1^iv^	−141.38 (9)	K1^iv^—O1—S1—C1	−107.68 (12)
O3—K1—O2—K1^iv^	15.65 (9)	K1^iii^—O1—S1—K1^iv^	−86.2 (6)
O4—K1—O2—K1^iv^	−53.99 (9)	Br1—N1—S1—O2	−177.66 (15)
O3^ii^—K1—O2—K1^iv^	146.08 (11)	Br1—N1—S1—O1	54.1 (2)
O2^ii^—K1—O2—K1^iv^	86.54 (16)	Br1—N1—S1—C1	−63.8 (2)
O1^ii^—K1—O2—K1^iv^	24.1 (3)	Br1—N1—S1—K1^iv^	129.71 (11)
S1^ii^—K1—O2—K1^iv^	66.47 (16)	C2—C1—S1—O2	60.1 (3)
O2—K1—O3—K1^iv^	−15.68 (9)	C6—C1—S1—O2	−117.8 (3)
O1^i^—K1—O3—K1^iv^	124.69 (15)	C2—C1—S1—O1	−178.6 (3)
O4—K1—O3—K1^iv^	89.55 (10)	C6—C1—S1—O1	3.6 (3)
O3^ii^—K1—O3—K1^iv^	−80.34 (16)	C2—C1—S1—N1	−53.3 (3)
O2^ii^—K1—O3—K1^iv^	−142.87 (10)	C6—C1—S1—N1	128.9 (3)
O1^ii^—K1—O3—K1^iv^	167.49 (10)	C2—C1—S1—K1^iv^	116.6 (3)
S1^ii^—K1—O3—K1^iv^	−167.32 (9)	C6—C1—S1—K1^iv^	−61.2 (3)
O2—K1—O4—K1^v^	−165.44 (7)		

Symmetry codes: (i) *x*+1/2, *y*, *z*−1/2; (ii) −*x*+1/2, −*y*, *z*−1/2; (iii) *x*−1/2, *y*, *z*+1/2; (iv) −*x*+1/2, −*y*, *z*+1/2; (v) −*x*+1, −*y*, *z*.

### Hydrogen-bond geometry (Å, °)

**Table:**

*D*—H···*A*	*D*—H	H···*A*	*D*···*A*	*D*—H···*A*
O3—H31···N1^iv^	0.80 (2)	2.16 (2)	2.937 (4)	164 (5)
O3—H32···Br1^vi^	0.80 (2)	2.83 (3)	3.574 (3)	156 (4)
O4—H41···N1^vii^	0.82 (2)	2.13 (3)	2.905 (4)	157 (5)

Symmetry codes: (iv) −*x*+1/2, −*y*, *z*+1/2; (vi) −*x*, −*y*, *z*; (vii) *x*+1/2, *y*, *z*+1/2.
